# Supplementary material for: Proportion of paediatric admissions with any stage of noma at the Anka General Hospital, northwest Nigeria
Source: PLoS Negl Trop Dis. 2023 Oct 27;17(10):e0011508. doi: 10.1371/journal.pntd.0011508 (PMC10645368; doi:10.1371/journal.pntd.0011508)
Supplement: Attachment — Submitted filename: Response letter_22.8.2023.docx [file pntd.0011508.s001.docx]

Dr. Elise Farley

Médecins Sans Frontières, Nigeria

Email: elisefarley@gmail.com

Phone: +27 79 5601 251

Dear Prof. Vinetz,

# I am writing in relation to the attached manuscript ‘Proportion of paediatric admissions with any stage of noma at the Anka General Hospital, northwest Nigeria’ for your continued consideration for publication in the PLOS NTD (PNTD-D-23-00848).

Thank you very much for the quick response and peer review of our submission. We appreciate the time you have taken to consider our manuscript. Please find below a response to each of the reviewers comments and attached a clean and tracked changes version of the ,manuscript.

We hope that you will continue to consider our request for publication favourably and look forward to your reply.

Warm regards,

On behalf of the author group,

Dr. Elise Farley (corresponding author)

Epidemiologist, Médecins Sans Frontières, Nigeria

**Black: Reviewer comments**

**Green: Author responses**

Reviewer #1: The objectives of the study are clearly articulated. The study design has some limitations which I will describe in the general comment section, regarding the population and sample. No ethical concerns.

Author responses: Thank you so much for your review and this comment.

Reviewer #1: The results are clear and figures/tables sufficient.

Author responses: Thank you.

Reviewer #1: The authors discuss some limitations. I recommend adding limitations in the general comments section. The public health relevance is clear.
Author responses: Thank you, we have responded to each of your suggestions below.

Reviewer #1: None

Author responses: Thank you.

Reviewer #1: Review PNTD-D-23-00848 PLOS Neglected Tropical Diseases 2023
Prospective study of children with any stage of noma admitted to a general hospital in northern Nigeria. With the very limited research on noma, this prospective study is welcome and significant. Few other studies include the stage of noma, especially the early stages when the disease is easily treated, preventing both mortality and morbidity. This research aimed to investigate the role of malnutrition in the epidemiology of noma, with a view to advising prevention strategies. The research emphasizes the need for oral exams of young children and improving tooth cleaning based on good breath smell and white teeth, which appeals to the children and their caregivers.

Author responses: Thank you for this summary.

The abstract is concise. I recommend adding the dates and length of the research study.

Author responses: This is a really helpful suggestion, thank you, we have made this change.

Limitations were the study length for only five months in 2021, during the pandemic and the location in the hospital.
Did this hospital have any records of children with the diagnosis of acute noma or any evidence of noma survivors?

Author responses: This is a good question, unfortunately there is no documented proof of later stage noma admissions at the Anka General Hospital, but several referrals have been made of patients being bought from the Anka General Hospital to the Noma Children’s Hospital. We have included this information in the introduction section of the manuscript.

What about the limitations of the research located at the hospital?

Author responses: Thank you, we have included this point.

Do children at this early stage visit the general hospital?

Author responses: Thank you, we have included this point.

What about extremely poor children, without access to health care and malnourished?

Author responses: Thank you, we have included this point.

Do you think this study was affected by the pandemic, limiting visits to the hospital?

Author responses: This is a good point, we have reflected on this in the manuscript.

Do you have any recommendations to suggest for future studies to reach vulnerable children outside the hospital setting?

Author responses: Thank you, we have included suggestions for future studies.

Noma is associated with children with chronic malnutrition, whereas this study targeted acute malnutrition. Did you investigate the proportion of children with chronic malnutrition (Height/age <-2SD, severe <-3SD)? Children with chronic malnutrition would not be admitted to the ITFC.

Author responses: Thank you for this suggestion, we have now included this in the analysis. This has provided clear evidence of the link between the early stages of noma and chronic malnutrition. Thank you for this suggestion, it is a valuable contribution to the manuscript.

Line 144, page 8
I don't understand this sentence, please clarify. Does this mean that you know the proportion of pediatric admissions with noma? Is the word "than" correct?
Author responses: We have clarified this in the manuscript.

Did you consider these two references?

Noma – a neglected disease of malnutrition
and poor oral hygiene: A mini-review
Wubishet Gezimu1 , Ababo Demeke2 and Abdissa Duguma1
SAGE Open Medicine
Volume 10: 1–5 2022
[sagepub.com/journals-permissions](http://sagepub.com/journals-permissions)
DOI: 10.1177/20503121221098110

Tickell KD, Walson JL (2016) Nutritional
Enteric Failure: Neglected Tropical Diseases and
Childhood Stunting. PLoS Negl Trop Dis 10(4):
e0004523. doi:10.1371/journal.pntd.0004523

Author responses: Thank you for these suggestions, we have included these references.


Thank you for your dedication to address the gaps in knowledge of noma epidemiology. The aim to improve the nutritional and  oral status of this vulnerable population and to recommend measures to prevent the development of noma are encouraged by your research and this publication. Thank you to PLOS NTDs for continuing to support noma research.

M. Leila Srour
MD FAAP MPH DTM&H

Author responses: Thank you so much for your review Dr Srour and for your continued support for noma survivors and efforts to prevent new cases of this terrible disease from developing. Thank you to PLOS NTD for being supportive of noma research.
